# Summary statistics for fossil spider species taxonomy

**DOI:** 10.3897/zookeys.192.3093

**Published:** 2012-05-08

**Authors:** David Penney, Jason A. Dunlop, Yuri M. Marusik

**Affiliations:** 1 Faculty of Life Sciences, University of Manchester, Manchester, M13 9PT, UK; 2 Museum für Naturkunde, Leibniz Institute for Research on Evolution and Biodiversity, Humboldt University Berlin, 10115 Berlin, Germany; 3 Institute for Biological Problems of the North, Magadan 685000, Russia; 4 Zoological Museum, University of Turku, FI-20014 Turku, Finland

**Keywords:** Arachnida, Araneae, palaeontology

## Abstract

Spiders (Araneae) are one of the most species-rich orders on Earth today, and also have one of the longest geological records of any terrestrial animal groups, as demonstrated by their extensive fossil record. There are currently around 1150 described fossil spider species, representing 2.6% of all described spiders (i.e. extinct and extant). Data for numbers of fossil and living spider taxa described annually (and various other metrics for the fossil taxa) were compiled from current taxonomic catalogues. Data for extant taxa showed a steady linear increase of approximately 500 new species per year over the last decade, reflecting a rather constant research activity in this area by a large number of scientists, which can be expected to continue. The results for fossil species were very different, with peaks of new species descriptions followed by long troughs, indicating minimal new published research activity for most years. This pattern is indicative of short bursts of research by a limited number of authors. Given the frequent discovery of new fossil deposits containing spiders, a wealth of new material coming to light from previously worked deposits, and the application of new imaging techniques in palaeoarachnology that allow us to extract additional data from historical specimens, e.g. X-ray computed tomography, it is important not only to ensure a sustained research activity on fossil spiders (and other arachnids) through training and enthusing the next generation of palaeoarachnologists, but preferably to promote increased research and expertise in this field.

## Introduction

**Figure 1.**
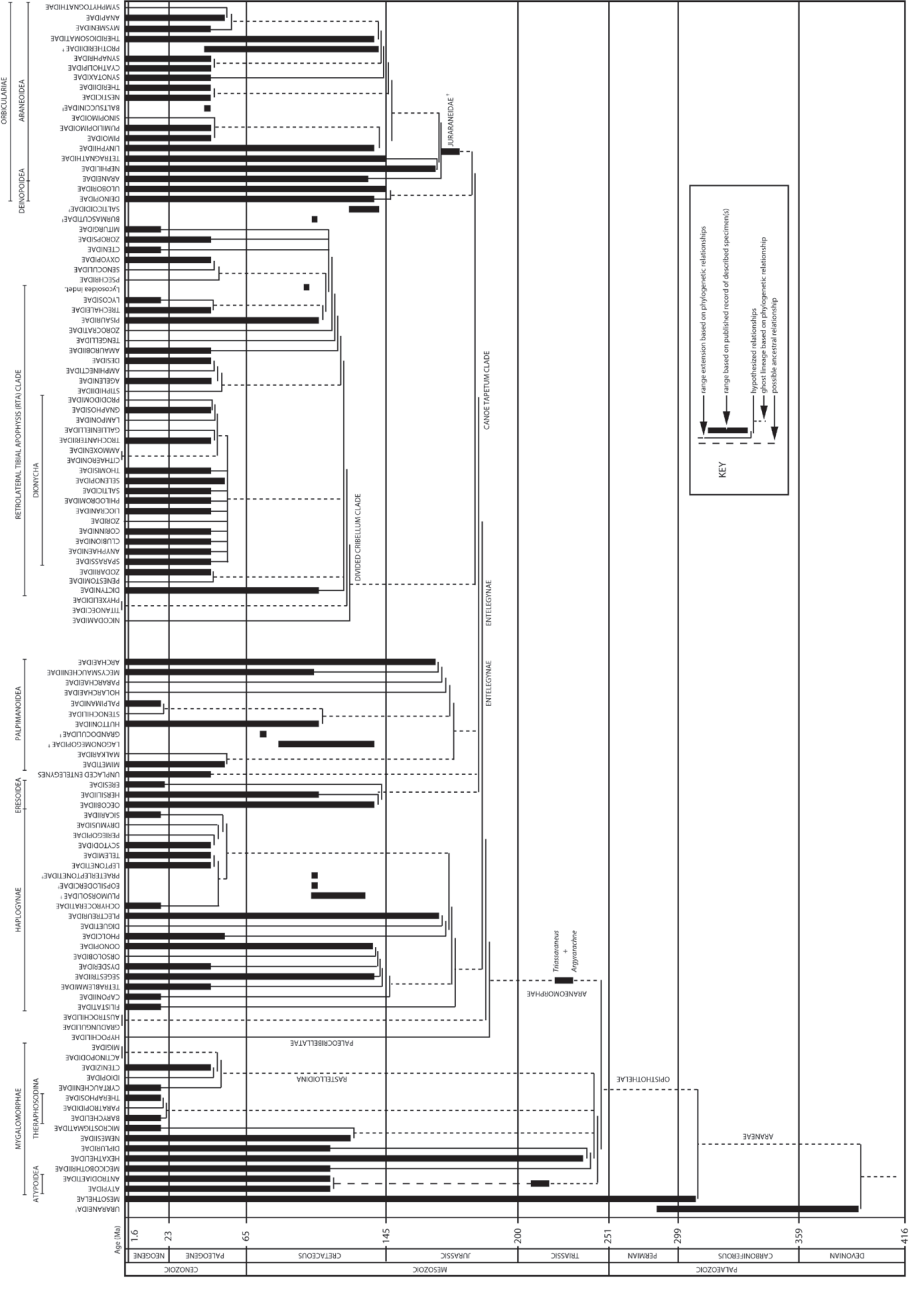
The evolutionary tree of spiders (updated from Penney and Selden 2011: data table 4) showing known ranges of spider families based on described fossils and predicted ranges of sister taxa based on their phylogenetic relationships (strictly fossil families inserted in approximate positions as per hypothesized relationships proposed in the literature, but not based on any cladistic analysis). Note that this is a highly dynamic figure, with known ranges and predicted ranges changing frequently as a result of new fossil discoveries, changes in phylogenetic hypotheses, revised dating of various deposits and even potentially through revised dating of geological periods and epochs. Researchers should check Dunlop et al. (currently 2012 and updated every six months) for the most recent data on the oldest fossils of each family and genus.

With 42,751 currently recognized extant species (Dunlop and Penney 2011; Platnick 2012), spiders (Araneae) are one of the most species-rich orders on Earth today and also have one of the longest geological records of any terrestrial animal groups as demonstrated by their extensive fossil record (Selden and Penney 2010; Penney and Selden 2011). They have the best documented fossil record of all arachnids (Dunlop and Penney 2012; Dunlop et al. 2012) with approximately 1150 described fossil spider species, representing around 2.6% of all described spiders (fossil and extant). Fossil spiders are most commonly found as inclusions in amber, where they usually represent 1.0–5.9% (3.2 ± 1.25) (Penney and Selden 2011: Table 6) of all inclusions, and in this mode of preservation they are usually autocthonous. Penney (2002) demonstrated that Dominican amber is biased towards preserving active spiders that lived on or around the amber producing tree, and Penney and Langan (2006) concluded that different ambers derived from resins that acted as a trap for spiders in the same way. However, fossil spiders also occur in sediments that would have accumulated in an aquatic setting, and in this case the vast majority are allocthonous, and accordingly they are much rarer than fossil spiders in amber. Spiders preserved in sediments are likely to have lived in close proximity to, or in webs suspended over the water body. Spiders appear to have been as diverse in the Eocene (e.g. Wunderlich 2004) as they are today and data are amassing to suggest a high diversity in the Cretaceous too. Evidence supporting this supposition derives not only from the fossils themselves, but also from the predicted range extensions of their related taxa based on their phylogenetic relationships (Figure 1).

One of the most valuable contributions that fossils can make towards modern studies of spider evolution is dating when groups or families first appeared. Fossils provide a minimum age for any given family (Figure 1) or genus and they have been used to calibrate molecular phylogenies (e.g. Dimitrov et al. 2011). However, molecular clock dates often predict splits between groups much further back than the evidence shown in the fossil record (e.g. Ayoub and Hayashi 2009). Of course older specimens may be discovered, but one of the most exciting recent developments has been the use of fossil data by molecular biologists to calibrate their molecular trees in attempts to determine when the major groups appeared and how the fossils fit into wider patterns of relationships.

New, significant amber deposits containing fossil spiders are being discovered frequently (e.g. Hand et al. 2010 – the first for Australia [an earlier record by Hickman 1957 actually refers to sub-fossilized copal]; Rust et al. 2010 – the first for India; Schmidt et al. 2010 – the first for Africa), and a wealth of significant new material from previously studied localities continues to be described (e.g. Penney 2009; Selden 2010; Selden and Huang 2010; Selden et al. 2011; Pérez-de la Fuente et al. in press; Dalla Vecchia and Selden in press). The application of synchrotron scanning (Saupe et al. 2012) and X-ray computed tomography (Penney et al. 2007, 2011; Bosselaers et al. 2010; McNeil et al. 2010) to fossil spiders has recently been used to extract new and additional morphological data from historical specimens too (Dunlop et al. 2011). This means that we can now revisit palaeospecies described more than 150 years ago in order to clarify their taxonomy within the present framework of spider systematics, using much closer taxonomic practices to those applied for extant taxa. These minimal preparation and non-destructive techniques also mean we can now visualize specimens in totally opaque amber (Perrichot et al. 2010) and because we can rotate and digitally dissect the 3D reconstructions (Penney et al. 2007, 2011; Dunlop et al. 2011; Saupe et al. 2012), specimens preserved in such a manner that the diagnostic features are obscured no longer hinder their taxonomic study. Previously, such specimens would have been set aside as impossible to work with. Here, we investigate some summary statistics relating to the history of fossil spider taxonomy and consider the implications of these for future research in this field.

## Methods

Data for numbers of fossil spider species (excluding subfossils in copal, peat cores and extant species collected from archaeological sites) were taken from Dunlop et al. (2012). Data for numbers of described extant spider species described for each year from 2001–2012 were taken from the ‘counts’ pages of Platnick’s online *World Spider Catalogs*. Only data for species currently considered to be valid were used. Data were plotted and examined qualitatively, although a best fit line was generated for the extant taxa. Various other metrics we considered may be informative with regard to the history of the description of new fossil spider species were also investigated. For comparable approaches using discovery accumulation curves for fossil arthropods – in this case trilobite genera – see e.g. Tarver et al. (2007). A similar curve for fossil scorpion species was also recently published by Legg et al. (2012: fig. 1).

## Results

**Figure 2.**
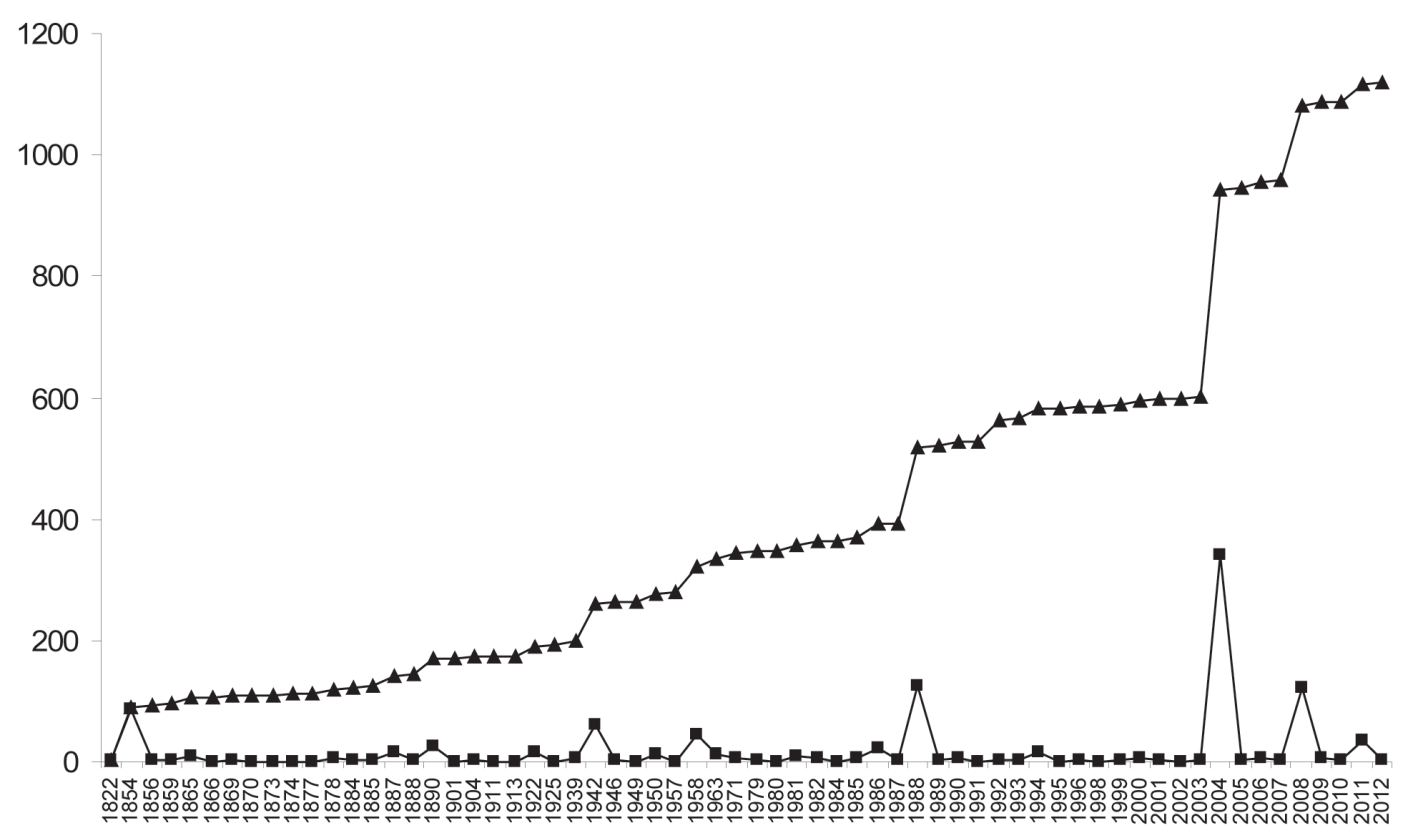
The numbers of described fossil spider species by year. Note that data for the 126 years where no fossil spider species were described are not included. Hence, the actual lull periods between peaks of activity are artificially shortened in this graph. For example, the period between the first described fossil spider in 1822 and the next data plot is actually 32 years. Squares = newly described fossil spider species, triangles = cumulative number of described fossil spider species. Data derived from Dunlop et al. (2012).

**Figure 3.**
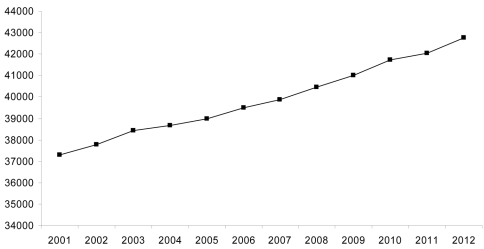
The cumulative number of newly described extant spider species this century. Data from Platnick (2001–2012). Total number of described extant species = 42,751 (Platnick 2012).

**Figure 4.**
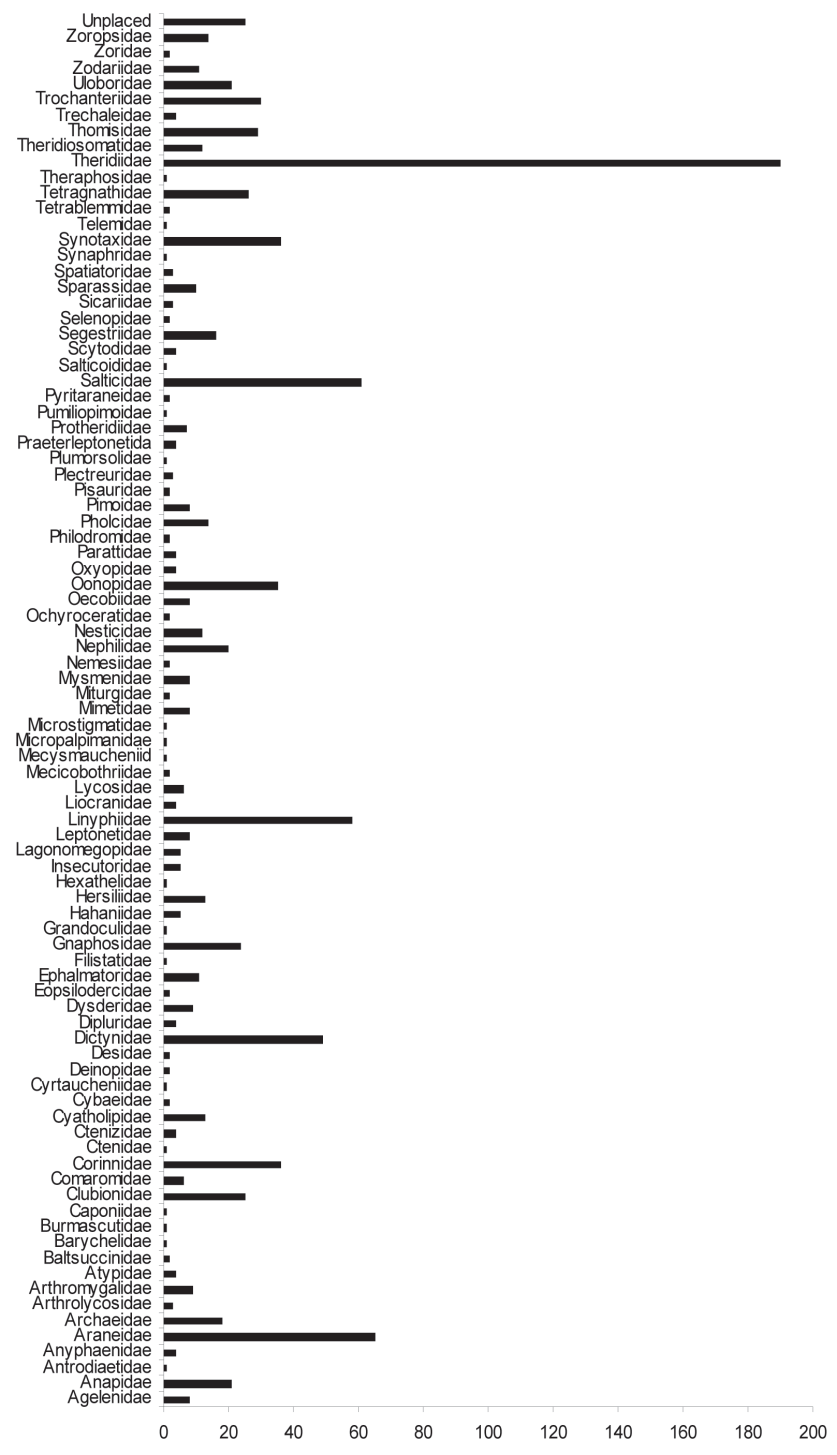
Number of fossil spider species per family (as currently assigned).

**Figure 5.**
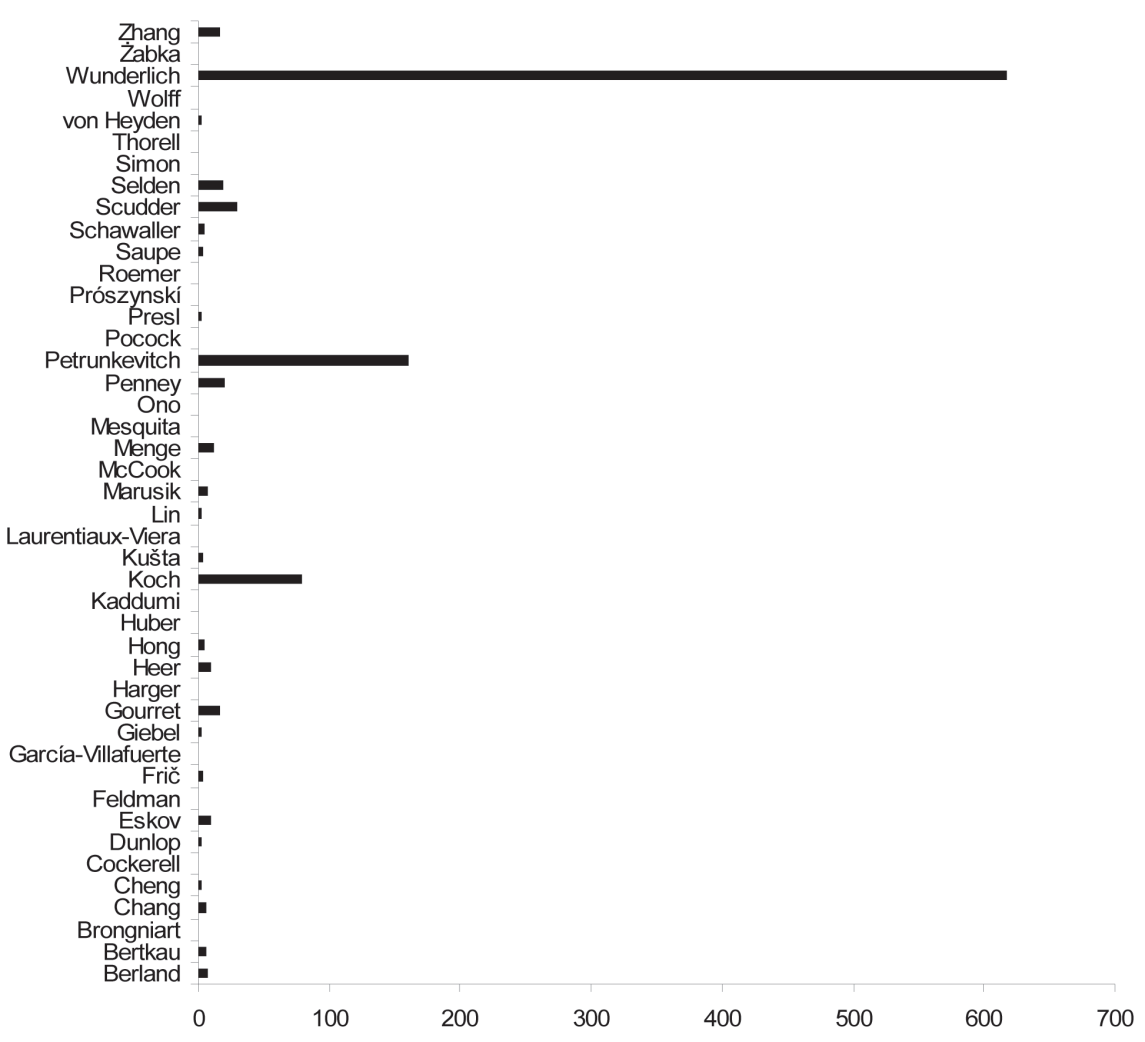
Number of fossil spider species described by different arachnologists. Only first authorship data are considered, so in reality some authors will have described more species than the value indicated.

Data for numbers of fossil spider species (excluding subfossils) are plotted in Figure 2 and data for extant species are plotted in Figure 3. The line of best fit (not illustrated) for the extant species data has a formula of y = 482.81x + 36738 (R^2^ = 0.992), suggesting an annual increment in the number of described extant species of approximately 480; based on a calculated mean of the actual data the value is 496 ± 162. The plot of the palaeontological data does not show a linear increase, but rather sporadic peaks interspersed with periods of little activity. The classification of fossil species within families is shown in Figure 4 and the numbers of fossil spider species per geological time period are shown in Figure 5.

## Discussion

Our research has focused on species as this tends to be the most informative unit of bio/palaeodiversity data; families are too few to allow any informative analysis on a broad scale, and genera are too idiosyncratically defined. Nonetheless, it is interesting to note that 70 (= 63%) extant spider families (including Comaromidae *sensu* Wunderlich 2011) have now been documented in the fossil record and there are an additional 18 strictly fossil families, the most recent described by Penney (2011). However, many of these extinct families are based on very few poorly preserved and/or juvenile specimens and require taxonomic scrutiny in order to confirm their validity (Penney and Selden 2006, 2011). Our data for the numbers of species described per year are actually under-representations because they do not include junior synonyms, *nomina nuda*, etc., a constraint applicable to both the fossil and extant data; nor do they include sub-fossils. However, such taxa are relatively few in the fossil record (Dunlop et al. 2012) so their exclusion will not have created any unrealistic trends.

It is evident from Figure 4 that some families are much more common as fossils than others, for example Theridiidae, Salticidae, Linyphiidae and Araneidae, and it is noteworthy that these represent four of the five most diverse spider families on the planet today. The fifth family is Lycosidae, which are ground dwellers and so are unlikely to be preserved in amber and most probably only evolved in the Miocene (Jocqué and Alderweireldt 2005). The reason for the high palaeodiversity in the aforementioned families is because they occur in various different deposits, whereas those with few described fossil species are often restricted to a single deposit. Penney and Langan (2006) compared the spider faunas of Baltic and Dominican ambers (which account for approximately 71% of described fossil spider species to date). There were more families (29) shared between the deposits than those that occurred in just one of the deposits (24 families restricted to Baltic, 15 families restricted to Dominican). Of further interest is that the shared families tended to be more diverse in each of the two deposits than the non-shared families. For example the average number of species per family for Baltic only families was 5.88, but for those families that also occurred in Dominican amber the average number of species was 12.44 for the Baltic fauna. Similarly, the average number of species for families specific to Dominican amber was 1.27 species per family whereas for those shared with Baltic amber the number of species was 5.17 for Dominican amber. In summary, 76% of all species belonged to families that were shared between both deposits and this value is most likely to rise, rather than fall, as a result of new fossil spider descriptions (e.g. Penney 2009; Penney et al. 2011, in press; Saupe et al. 2010; Wunderlich 2008, 2011). These data are based on relatively young Tertiary fossils, so their similarity to the extant fauna should not be a great surprise. If we had a similar number of fossil spider species described from the Mesozoic then we could expect a rather different pattern, particularly as two of the families that are most diverse in the Tertiary (Salticidae and Theridiidae) are currently unknown from the Mesozoic and probably evolved (or at least underwent their major diversification) following the end-Cretaceous extinction event. It must also be remembered that the spider fossil record is heavily biased towards amber, so the observed palaeodiversity is an artefact of sampling and so is not truly representative of what existed in the past.

**Figure 6.**
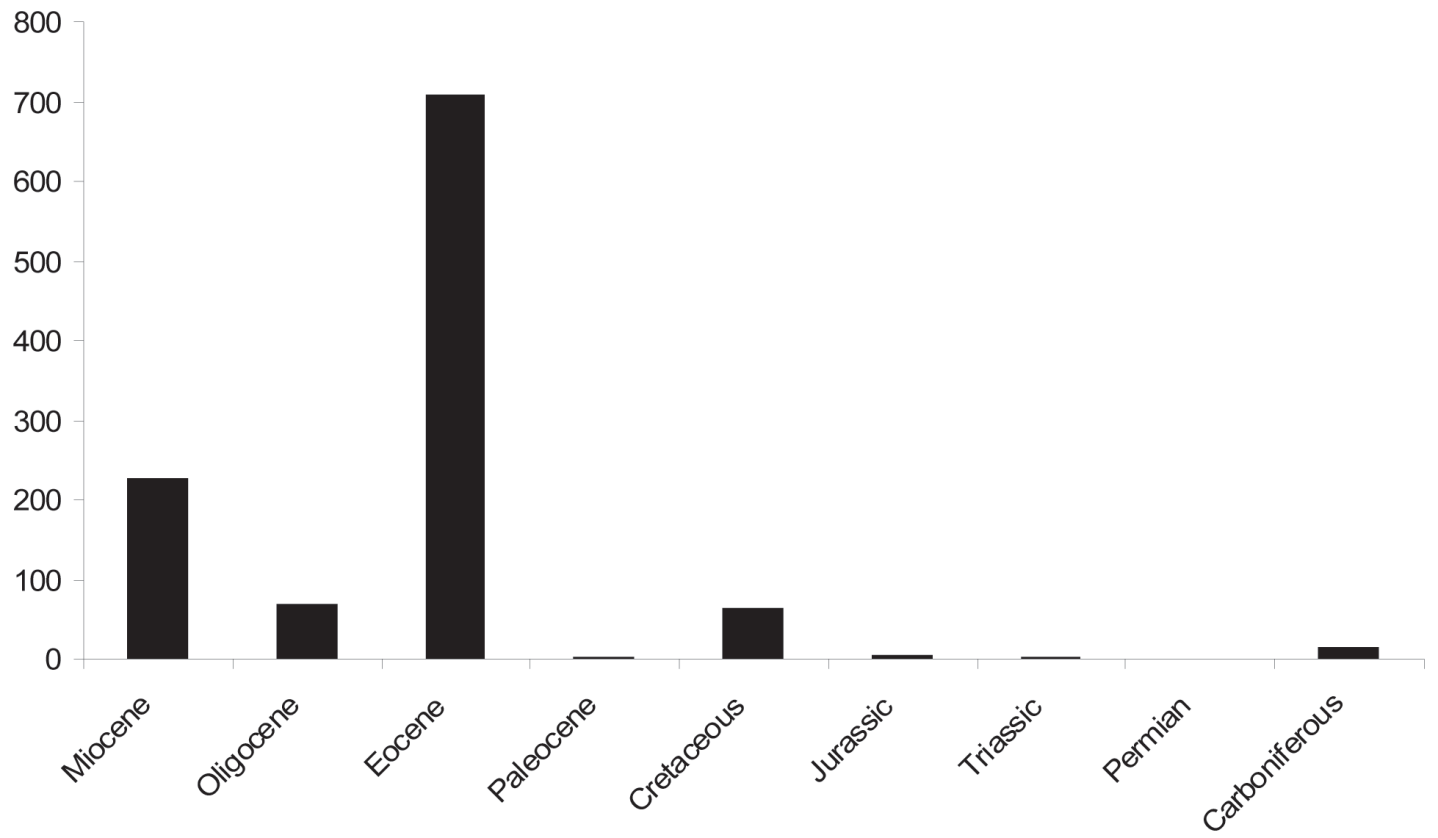
Number of fossil spider species described from each geological period. The Paleogene Period has been broken down into its various Epochs (Paleocene, Eocene and Oligocene) in order to show the spread of data; the Neogene Period is represented only by the Miocene Epoch because Pleistocene sub-fossils have not been included.

New extant spider species are described every year, but this is not so for fossils. In our palaeodata there are 126 years in which no fossil spider species were described. These are omitted from the graphs, so the actual lull periods between peaks of activity are artificially shortened in Figure 3. For example, the period between the first described fossil spider by Presl (1822) and the next data point (Koch and Berendt 1854) is 32 years. Since 1822 we have 64 data points for years with described fossil species, equating to approximately 33% of years with newly described fossils. There is no linear pattern to the increment of new fossil spider species. The peaks in Figure 3 represent the publication of monographs that focus on particular deposits (e.g. Koch and Berendt 1854; Petrunkevitch 1942, 1958; Wunderlich 1988, 2004, 2008, 2011) and most of these, and consequently the authorship of described fossil spider species, can be assigned to a limited number of authors (Figure 5). In total, 44 researchers have described valid fossil spider species, with 54% of the names assigned to a single author (J. Wunderlich), who is still publishing on the topic. These data refer only to the first authorship of a published taxon, so in reality there are actually more species attributed to individual authors than Figure 5 suggests (as a result of co-authored taxa). It also means there is a descriptive bias to particularly productive deposits and hence geological periods (Figure 6) (see also Saupe and Selden 2011). Indeed, 652 fossil spider species have been described to date from the Baltic amber deposits, representing approximately 57% of all named fossil spider species. This is followed by Dominican amber with 164 named species, representing approximately 14%. Other than the Eocene Florissant Formation and Miocene Bitterfeld amber, both of which have 46 described fossil spider species, all other fossil deposits currently have 25 or fewer described fossil spider species, and in most the number of described taxa rarely exceeds five (Dunlop et al. 2012). However, work on Baltic amber spiders has spanned almost two centuries, whereas the first spider from Dominican amber was not described until 1981 (Penney 2008).

It should be noted that the holotypes of many of the older species names – e.g. the Florissant specimens described by Scudder (1890) and Petrunkevitch (1922) – require taxonomic revision in order to confirm their status. Many of the early Baltic amber taxa (e.g. those of Koch and Berendt 1854) were treated in the recent monographs by Wunderlich (2004, 2008).

Data for extant taxa showed a steady linear increase of approximately 500 new taxa per year over the last decade, reflecting a rather constant research activity in extant spider taxonomy by a large number of scientists, which can be expected to continue. The results for the description of fossil species were very different, with peaks of new species descriptions followed by long troughs indicating short bursts of research by only a few authors, often with a long hiatus in between. Were these data to represent patterns within natural populations, one would consider the latter to be at considerable risk of extinction. Given the frequent discovery of new fossil deposits containing spiders, a wealth of new material coming to light from previously worked deposits, and the application of new imaging techniques in palaeoarachnology that allow us to extract additional data from historical specimens, e.g. X-ray computed tomography, it is important not only to ensure a sustained research activity on fossil spiders (and other arachnids) through training and enthusing the next generation of palaeoarachnologists, but preferably to promote increased research and expertise in this field.
